# Feasibility and Effectiveness of Portable/Remote Heart Rate Variability (HRV) Biofeedback Training in Treating Stress and Mental Health Difficulties: A Systematic Review

**DOI:** 10.1007/s10484-025-09751-9

**Published:** 2025-12-09

**Authors:** Sophie Wilson, Alisia-Roberta-Lorelai Maciu, Katie Ashcroft

**Affiliations:** 1 Royal Holloway, University of London, London, UK; 2 Central and North West London NHS Foundation Trust, London, UK; 3 University College London (UCL), London, UK

**Keywords:** Systematic review, Heart rate variability, Biofeedback, Portable biofeedback, Remote biofeedback, Stress management, Anxiety, Depression

## Abstract

Understanding the mind–body relationship is increasingly important in healthcare. Dysregulation of the autonomic nervous system (ANS) has been linked to both physical and mental health issues. Heart rate variability biofeedback training (HRVBt), which targets ANS regulation, is gaining popularity and is becoming more portable with advancing technology. While meta-analyses have shown that HRVBt can improve psychological and physiological outcomes, no review has specifically examined portable/remote HRV biofeedback training (P/R-HRVBt). Given the digital transformation of healthcare, this review aimed to evaluate the feasibility and effectiveness of P/R-HRVBt for reducing stress and subclinical anxiety and depression, and to consider its clinical implications. Three databases (PubMed, PsycINFO, Web of Science) and reference lists were searched. Eligible studies were written in English, used P/R-HRVBt, and quantitatively assessed feasibility or effectiveness on psychological outcomes or physiological stress markers in adults experiencing stress or subclinical anxiety and depression. Thirteen studies met the inclusion criteria. Quality was appraised using the Quality Assessment Tool for Quantitative Studies. All studies received a global ‘weak’ rating, though four achieved ‘moderate’ quality when blinding was excluded. Overall, P/R-HRVBt was found to be feasible. All studies reported improvements in at least one psychological or physiological outcome, though heterogeneity in study design, measures, and effect sizes limited conclusions. Further high-quality research is warranted. Nonetheless, findings suggest that P/R-HRVBt may be comparable to established interventions and offers a promising alternative for improving psychological and physiological health.

## Introduction

There is growing evidence that physical and mental health difficulties are associated with dysregulation in the autonomic nervous system (ANS). The ANS is responsible for regulating involuntary bodily functions such as heart rate, respiration, and digestion (Thayer & Brosschot, 2005). As a result, understanding the mind–body relationship is becoming an increasingly important topic for healthcare professionals and is also receiving interest from the general public (Bandealy et al., 2021). Interventions that have been developed to try and improve regulation in the ANS have shown promise in improving mental and physical health outcomes, from general well-being to mental or physical health conditions (Grasser & Marusak, 2023). These interventions have been considered an alternative or complementary approach to traditional methods of psychotherapy and medication (Burnett-Zeigler et al., 2016). They are also relatively low-cost and have the potential to provide long-term benefits beyond initial training, such as increased resilience, stress-coping skills, improved sleep quality, and protection against adverse experiences (Burnett-Zeigler et al., 2016; Grasser & Marusak, 2023; Laird et al., 2018).

The use of digital interventions in healthcare is also becoming increasingly popular. The high prevalence of mental health difficulties and the recent acceleration of remote care as a result of the COVID-19 pandemic have led to a surge in digital interventions (Moshe et al., 2021; Nochaiwong et al., 2021). The Department of Health and Social Care (DHSC) and NHS England are also prioritising digital transformation, recognising the use of technology platforms such as smartphones, tablets, and computers to help reduce pressure on the overstretched workforce, address disparities in access and outcomes, and empower individuals to self-manage their difficulties (Department of Health & Social Care, 2022). Further, digital psychological interventions have been shown to be an effective and practical model of care for the growing prevalence of mental health difficulties, with treatments being delivered directly into people’s hands with high-fidelity and no-to-low human resources (Andersson & Titov, 2014).

One approach that considers the mind–body relationship is Heart Rate Variability Biofeedback training (HRVBt). It has gained attention as a treatment for mental and physical health difficulties and is becoming more portable with the advancement of technology.

### Heart Rate Variability

Heart rate variability (HRV) refers to the variation of time between heartbeats, which is called R-R intervals (Thayer & Brosschot, 2005). It is a recognised biomarker of the ANS and emotion regulation (Edwards & Pinna, 2020; Pizzoli et al., 2021).

The ANS plays a crucial role in managing emotions and maintaining a balance of many bodily functions, which include heart rate, digestion, and respiratory rate, ensuring optimal body functioning. It comprises the sympathetic nervous system (SNS) and the parasympathetic nervous system (PNS). The SNS, referred to as the ‘fight or flight’ response, prepares the body for action by increasing an individual’s heart rate, blood pressure and breathing rate; in doing so, it shortens the interval between heartbeats (McCorry, 2007). The PNS, known as the ‘rest and digest’ response, tries to counteract the SNS and actively tries to slow down these processes and promote longer intervals between heartbeats to help restore the balance of the ANS (Weerdmeester et al., 2020; Yu et al., 2018). The SNS and PNS work together, and usually when one system is activated, the other is suppressed. For example, when faced with a stressor, the SNS activates to prepare the body for action and once the stressor is over, the PNS takes over to calm the body down. This interplay between the SNS and PNS is crucial for effective emotion regulation (Edwards & Pinna, 2020). When the SNS and PNS are working well, the body can effectively respond to stress and regulate emotions and bodily functions. However, prolonged periods of stress can dysregulate the ANS, which can be detrimental to overall health and well-being (Alvares et al., 2016; Thayer & Brosschot, 2005).

HRV serves as a marker of the ANS and reflects the interplay between the PNS and SNS activity. Low HRV signifies increased sympathetic activity (Reiner, 2008), and is associated with various mental health difficulties, including anxiety and depression (Thayer & Brosschot, 2005). High HRV, on the other hand, indicates parasympathetic activity, which predicts greater mental health resilience and better emotional regulation (Edwards & Pinna, 2020; Perna et al., 2020). Overall, HRV reflects the interplay between the PNS and SNS activity, and interventions that effectively target HRV are warranted.

#### HRV Indices Definitions

HRV indices are measures that assess the ANS. In this review, the following are referenced:

- Standard Deviation of Normal-to-Normal Intervals (SDNN) measures the overall variability of heartbeats, for example, the fluctuation in the time between each heartbeat over a length of time (Pham et al., 2021).
- Root Mean Square of Successive Normal-to-Normal Interval Differences (RMSSD) measures the variability between each heartbeat, for example, it looks at the change in the time between each heartbeat from one beat to the next (Pham et al., 2021).
- Low Frequency (LF) and High Frequency (HF) represent different frequency ranges in the HRV signal. LF is associated with PNS and SNS activity, while HF is more related to PNS activity (Pham et al., 2021).

### HRV Biofeedback Training

HRVBt is a non-invasive tool designed to help individuals improve and restore the autonomic regulation of their heart rate (HR) by directing slowed-paced breathing (Lehrer, 2003). It involves displaying raw signals of HRV to individuals through auditory or visual means while they practice slow-paced breathing (Lehrer et al., 2000). When receiving this information, individuals can consciously adjust their breathing and learn to regulate their HR. Breathing at a slow and steady rate, such as six breaths per minute, slows the HR down and activates the PNS, thus increasing HRV by stimulating autonomic reflexes (Kim, 2023; Lehrer et al., 2003).

Traditionally, HRVBt required significant professional training and expertise. The equipment needed was difficult to operate, bulky, and expensive, and it mainly took place in laboratories or clinic spaces (Austad & Gendron, 2018). However, there has been interest in making HRVBt more accessible, with the development of portable and remote devices to overcome the limitations of traditional equipment in terms of cost and portability. Portable HRVBt devices are small handheld, wearable gadgets or can be smartphone applications. Remote devices are similar to portable devices but also allow healthcare professionals to monitor individual usage. These technological advances appeal to today’s society, with recent generations increasingly using technological platforms to boost their mental and physical health, including smartwatches, which have extended beyond healthcare.

### Current Knowledge and Gaps

There have been several meta-analyses conducted on HRVBt as an intervention for mental health. HRVBt has been found to significantly reduce self-reported stress and anxiety, as well as improve depressive symptoms across different psychophysiological conditions in adult samples (Goessl et al., 2017; Pizzoli et al., 2021). Similarly, Lehrer et al. (2020) performed a systematic review and a meta-analysis of HRVBt studies across many physiological and psychological conditions. They found that effect sizes were larger for the HRVBt group in comparison to the non-active than active control groups, although significant for both. This review also supported using HRVBt as a complementary treatment alongside traditional therapy.

Moreover, following the increased use of digital interventions and the up-and-coming use of more portable technology for HRVBt, ter Harmsel et al. (2021) examined the effectiveness and feasibility of using portable HRVBt devices in (addition to) treating emotion regulation difficulties. They found positive effects on self-reported stress-related psychological measures and significant improvements in physiological measures. Overall, these reviews suggest that HRVBt can effectively improve psychological and physiological outcomes.

However, it is important to note that the studies included in these reviews used different delivery methods, such as face-to-face sessions with more traditional HRVBt equipment, portable devices used in face-to-face sessions or remotely, or a combination of both. While the reviews concluded the overall efficacy of HRVBt interventions, it is difficult to assess the effectiveness of each delivery modality and determine if factors such as therapist contact or treatment protocol contribute to the results of HRVBt. Evaluating the effectiveness of different delivery methods may help to identify what works best for each individual and inform treatment delivery.

There are also many studies looking at the effectiveness of integrating the use of remote and portable HRVBt into existing treatments, such as Cognitive Behavioural Therapy (CBT), Dialectical Behavioural Therapy (DBT) and, more recently, Acceptance and Commitment Therapy (ACT) (Economides et al., 2020; Gevirtz, 2015). However, to the best of the author’s knowledge, no review has specifically focused on portable HRVBt as a stand-alone remote intervention. Given that the DHSC (2022) have identified digital transformation of health and social care as a priority, it would be beneficial to systematically review the literature, provide an overview of the current evidence of portable HRVBt as a remote intervention, evaluate its feasibility and effectiveness as a treatment modality, and consider possible clinical implications.

### Aims of the Review

This systematic review differs from previous reviews that have examined the effectiveness of HRVBt on psychological and physiological outcomes, as those reviews included studies that differed in the delivery of HRVBt. In contrast, this review specifically intends to examine studies investigating the effectiveness of using portable HRVBt devices as a remote intervention. This means that the participants included in these studies must have independently used the HRVBt device with minimal support from a therapist in their own environment. In this review, this type of HRVBt will be referred to as ‘portable/remote HRV Biofeedback training (P/R-HRVBt)’. This review aims to determine whether P/R-HRVBt is feasible and whether it effectively reduces mental health difficulties and stress levels.

## Method

### Protocol

This review followed the Preferred Reporting Items for Systematic Reviews and Meta-Analysis guidance (PRISMA; Moher et al., 2009) and the Synthesis Without Meta-Analysis in Systematic Reviews (SWiM; Campbell et al., 2020). A protocol and research strategy were developed to guide the process. The protocol was registered on the PROSPERO International Prospective Register of Systematic Reviews on 11th August 2023 (crd.york.ac.uk/prospero, registration number CRD42023449296).

### Eligibility Criteria

The eligibility criteria for the present review was developed using the PICOS tool (Miller & Forrest, 2001) and are outlined in Table 1.

**Table 1:** Eligibility criteria

	Inclusion criteria	Exclusion criteria
Population	Use samples from a clinical mental health setting or the general population, with adults aged 18 or over who have either self-identified with stress or mental health difficulties or who meet the diagnostic criteria for a mental health condition. Studies that included participants receiving medication as part of their treatment as usual (TAU) were eligible as populations recruited from a clinical mental health setting are likely to be receiving treatment.	Studies that include any participants who are younger than 18.
Intervention	Include P/R-HRVBt. HRVBt must be self-led and completed in the participant’s environment to ensure the intervention is fully portable and remote. The initial orientation session with a therapist or HRVBt trainer can occur in person as participants need to be set up with the device and taught how to practice HRVBt.	P/R-HRVBt being completed in a lab-based setting, in the presence of a therapist or HRVBt trainer, or paired with another active treatment (e.g., mindfulness or CBT), as this indicates the intervention was neither portable/remote nor a standalone intervention.
Comparison	There are no restrictions on comparators. Variations in protocols, follow-up length, intervention comparators, and studies without a comparison or control group were eligible for inclusion.	
Outcome	Assess the feasibility or effectiveness of P/R-HRVBt. Feasibility outcomes will assess if it is possible to implement an intervention in the intended setting and population (Gadke et al., 2021). For studies evaluating feasibility, at least one of these factors must be reported: P/R- HRVBt completion rate, adherence, dropout rates, or recruitment rates, which aligns with a proposed feasibility framework (Gadke et al., 2021). Effectiveness will assess whether the intervention can be performed under ‘real-life’ conditions (Singal et al., 2014) and measure either psychological outcomes or objective markers of physiological stress responses.	Physical health conditions as the targeted problem, as the review is looking at the effect of the intervention on mental health difficulties and stress as the targeted outcome
Study Design and Types of Study	Have a quantitative design such as assessment at pre-post intervention, clinical trials, single- case experimental designs (SCEDs), follow-up studies, and treatment outcome studies Studies must be available in English. There are no restrictions on publication date.	Grey literature, including reports, government documents, book chapters or reviews, were not included due to the potential lack of control, for example, not being in a peer-reviewed process. Protocols, single-subject case studies and qualitative studies.

### Search Strategy

The search terms were generated through initial scoping searches and considering terms used by past relevant systematic reviews. These terms were reviewed and discussed with the Royal Holloway University of London librarian, who has expertise in systematic reviews. Table 2 shows the search terms and Boolean operators used.

**Table 2:** Search terms used for the systematic review of databases

	Search terms
HRV Biofeedback	‘heart rate variability biofeedback’ OR HRVB OR biofeedback OR ‘HRV biofeedback’ OR ‘sinus arrhythmia’ OR ‘cardiac coherence’ OR ‘paced breathing’ OR ‘resonance frequency feedback’ OR ‘RSA biofeedback’ OR RFF OR biofeedback
(AND) Remove/portable device	Home OR ‘home-based’ OR ‘home based’ OR homebased OR ‘home training device’ OR ‘remote device’ OR wearable OR smartphone OR ‘smart phone’ OR device OR ‘portable device’ OR ‘stress eraser’ OR emWave OR internet OR digit* OR online OR ‘digital portal’ OR ‘online platform’ OR remote* OR app* OR ‘mobile technology’ OR ‘mobile device’ OR phone OR ‘mobile phone’ OR ‘handheld device’ OR ‘digital device’ OR ‘online intervention’
(AND) Stress or Mental Health Difficulty	‘alcohol addiction’ OR ‘alcohol craving’ OR ‘alcohol dependence’ OR alcoholism OR anger OR anxiety OR anxi* OR ‘anxiety disorder’ OR ‘performance anxiety’ OR borderline OR dementia OR depressi* OR ‘drug abuse’ OR ‘drug craving’ OR ‘eating behaviour’ OR ‘food craving’ OR gambling OR hyperactivity OR ‘major depression’ OR ‘obsessive compulsive disorder’ OR ocd OR panic OR ‘panic disorder’ OR agoraphobia OR ‘social phobia’ OR ‘social anxiety disorder’ OR ‘social anxiety’ OR ‘performance anxiety’ OR ‘generalised anxiety’ OR gad OR ‘perinatal depression’ OR ‘phobia’ OR psychosis OR ‘schizoaffective disorder’ OR schizophrenia OR stress OR ‘substance use disorders’ OR ‘suicidal behaviour’ OR ‘test anxiety’ OR ‘mental health’ OR ‘mental disorder’ OR ‘mental health disorder’ OR ‘post-traumatic stress’ OR ‘post-traumatic stress disorder’ OR ‘posttraumatic stress disorder’ OR stress OR ‘acute stress’ OR ‘personality disorder’ OR psycho* OR schizo*
(AND) Outcome	Acceptability OR accept* OR engagement OR efficacy OR effect* OR effective* OR ‘treatment effectiveness’ OR ‘outcome’ OR ‘clinical efficacy’ OR feasibility OR ‘feasibility study’
(NOT)	child* OR youth OR adoles* OR teenage*

Relevant studies were identified by searching the following three electronic databases: PsychINFO, which contains research specific to behavioural and social science; Web of Science, which contains research across a range of scientific fields; and PubMed, providing a wider pool of research in medicine and health. All searches utilised the ‘Advance Search’ function, and the search terms were applied to titles and abstracts with the limiters of ‘English only’, ‘Humans’ and ‘Adults 19 + ’ for PubMed; ‘English only’, ‘Adulthood (18 years & older)’, ‘Academic Journals’, and ‘Peer-reviewed’ for Psych Info; and ‘English only’ and ‘Article’ for Web of Science. The papers could have been published at any time up until 2nd January 2024. The initial search of each database was run on the 16th August 2023 and then repeated on the 2nd January 2024 to acquire any papers published between the initial search and write-up. A final search was performed on the 22nd May 2025 (and refined following feedback) to acquire any new papers published between January 2024 and May 2025.

### Selection Process

**Fig. 1:**
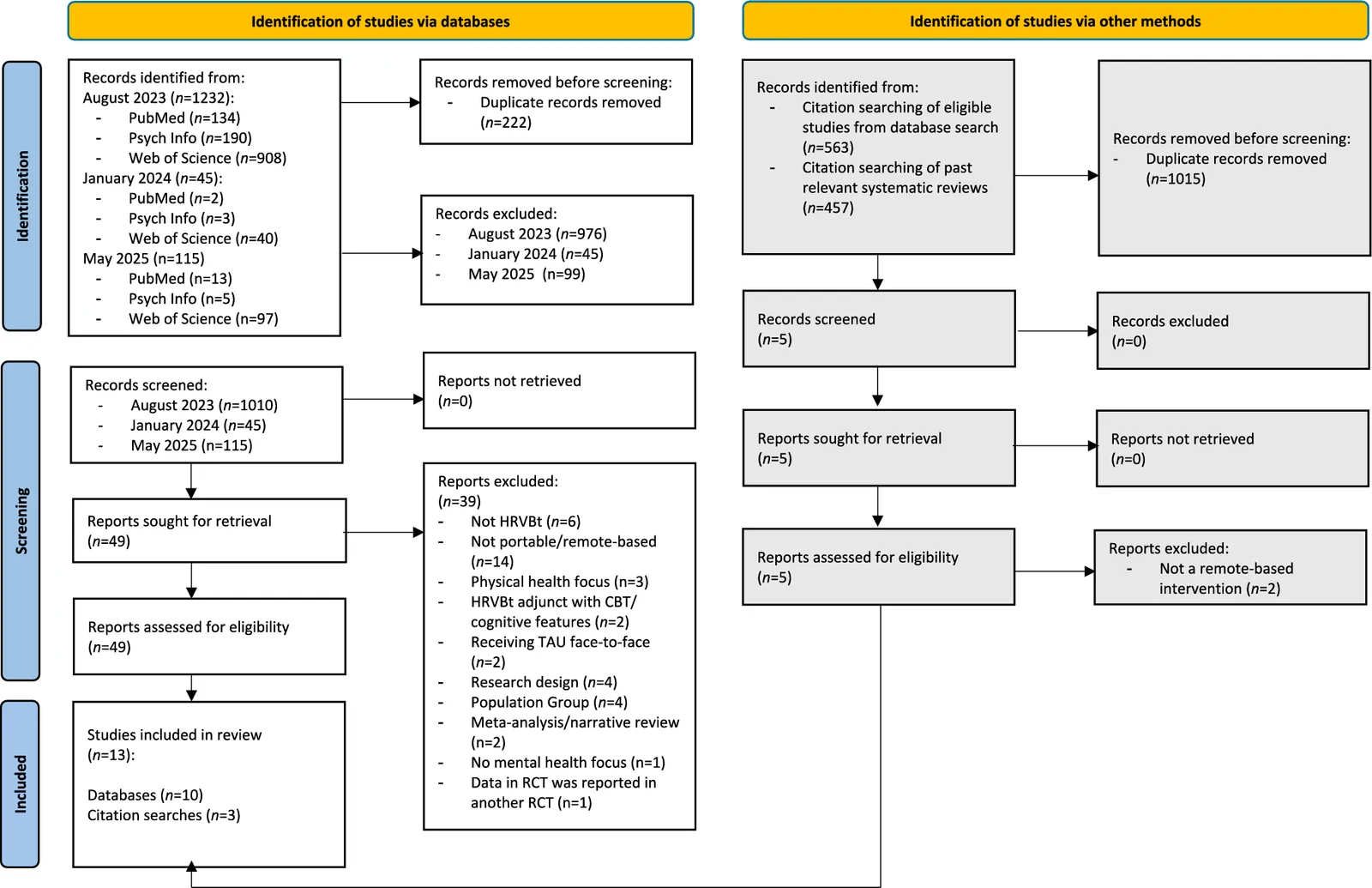
PRISMA diagram

Figure 1 shows the selection process used to identify eligible studies. The search results were exported to Zotero, a referencing management software, and screened for duplicates. In the initial search, 1232 references were retrieved from PubMed (*n* = 134), Psych Info (*n* = 190), and Web of Science (*n* = 908). After removing the duplicates, 1010 remaining articles were exported to Rayyan (Ouzzani et al., 2016), an online web app designed to manage systematic review screening and management.

Titles and abstracts of these remaining articles were screened for eligibility by the primary investigator (PI), and 976 were excluded due to not meeting the inclusion criteria. The full texts of the remaining 34 articles were screened against the eligibility criteria, and 25 were excluded.

During the selection process, two RCTs (de Bruin et al., 2016; van der Zwan et al., 2015) were found to use the same participant sample. The results of the primary outcomes were described in the paper by van der Zwan et al. (2015), and so this was selected as the ‘core paper’. The other study was used as a supplementary source of information. The PI based this decision on best practice guidance detailed in the following reference: Boland et al. (Ed.) (2017). An additional exclusion criterion was applied during the selection process to exclude studies targeting substance misuse disorders due to their known suppression of HRV (Moon et al., 2023; Ralevski et al., 2019).

A citation search was conducted to minimise the risk of missing relevant studies. References from all included papers and past systematic reviews were imported into Zotero and systematically screened, which led to the identification of three additional eligible studies. This supplementary step enhanced the completeness and thoroughness of the review process. The search strategy was repeated in January 2024 and May 2025 to identify any eligible new publications; 45 new papers were found, but none were identified as eligible, so they were excluded. During the May 2025 search, 115 new papers were screened for eligibility. Of these, 99 were excluded, and the remaining papers underwent further screening.

In total 13, studies satisfied the eligibility criteria and were included in the data analysis. An independent reviewer screened the abstracts of 10% of the original 1010 studies against the eligibility criteria to check for consistency in the selection process. There was a percentage agreement of 79.5% (K = .795), suggesting good interrater reliability. There was only one discrepancy, which was resolved through discussion.

### Data Extraction

The PI extracted the data from each eligible study using an Excel spreadsheet and referred to the inclusion criteria. Nine authors were contacted via email for additional information. If there was no response within two weeks of the email, missing data was recorded as ‘not reported’. Two authors replied with the requested information.

The study characteristics that were extracted included: (a) first author, year of publication, and country; (b) design (as defined by the quality assessment tool); (c) setting, inclusion criteria, and recruitment strategy; (d) sample characteristics, including sample size, age (mean, SD and range) and gender; (e) intervention characteristics, including type of P/R-HRVBt device, duration and frequency of intervention, orientation plan and adherence strategies; (f) comparator/control characteristics; (g) intervention duration and; (h) study completion rate.

To address the research question regarding the feasibility and effectiveness of P/R-HRVBt, the following information for each study was extracted: (a) outcome measures used and time points of data collection; (b) feasibility data, including P/R-HRVBt completion rate, recruitment rate, adherence, and dropout rate; (c) within group effects pre-to-post intervention and between-group effects post-intervention, in terms of reported statistical significance and (reported or calculated) effect sizes.

### Quality Assessment

The PI used the Quality Assessment Tool for Quantitative Studies developed by the Effective Public Health Practice Project (EPHPP; Thomas et al., 2004) to assess the quality of the included studies and the potential risk of bias. The EPHPP appraises quantitative studies within the public health sector and has shown strong content validity and test–retest reliability (Thomas et al., 2004). The tool comprises eight domains: selection bias, study design, confounders, blinding, data collection methods, withdrawals and dropouts, intervention integrity and analyses. A rating of ‘weak’, ‘moderate’, or ‘strong’ is allocated for six of the eight domains, resulting in a global quality rating for the overall study. A study is of ‘weak’ quality if two or more ‘weak’ ratings are acquired. Moderate studies will have acquired one ‘weak’ rating, whilst a global score of ‘strong’ is given to studies without any ‘weak’ scores.

An independent reviewer assessed the quality of 20% of the included studies. Studies were randomly selected using a random number generator. Interrater concordance was 100% (Cohen’s K = 1.00), suggesting good interrater reliability. There were no disagreements over ratings.

### Data Synthesis

The heterogeneity of the included studies regarding design, comparators and outcome measures meant that a meta-analysis was not appropriate. The data was therefore synthesised narratively using the guidance from PRISMA (Moher et al., 2009) and SWiM (Campbell et al., 2020). The analysis of the selected papers considered the feasibility and effectiveness of P/R-HRVBt. To achieve this, the included studies were grouped based on study characteristics and results and presented in tabular and narrative summaries. Studies rated as having lower concerns for quality bias (better quality assessment ratings) are presented first in the tables. The narrative summaries grouped effectiveness outcome data according to psychological outcomes (depression, anxiety, stress, well-being, and other psychological effects) and physiological outcomes (HRV and other physiological stress markers).

#### Feasibility

To determine the general feasibility, recruitment rates were calculated as the percentage of eligible individuals who consented to participate in the study, and the overall study completion rates were calculated as the percentage of participants who finished each study.

To assess the feasibility of the P/R-HRVBt intervention, completion rates were calculated as the percentage of participants in the intervention group who commenced and finished the study. To understand how many people adhered to the P/R-HRVBt protocol, adherence rates were calculated as the proportion of participants who completed the intervention protocol as defined by the researchers. To understand how many people finished the intervention, dropout rates were calculated as the percentage of participants who started the study but did not complete it. In instances where feasibility data was not described, this was calculated when provided data permitted.

#### Effectiveness

Statistical significance (*p*) and effect sizes (ES) were reported for all outcomes. Cohen’s *d* or Eta squared (η2) was reported for parametric tests, while Rosenthal’s *r* was used for non-parametric tests or when parametric test assumptions were violated (Cohen, 1988; Rosenthal, 1994). Eta squared is typically reported in studies using ANOVA. In the narrative summaries, the effect sizes were reported as presented by the studies for consistency of interpretation. Hedges’ *g* served as a secondary effect size to account for smaller sample sizes (Hedges et al., 2012) and were reported alongside Cohen’s *d* in the results table. Hedges’ *g* and Cohen’s *d* (when not provided) were calculated using means, standard deviations, and sample sizes with an Excel tool founded by Beckham (2016). Table 3 outlines effect size classifications.

**Table 3:** Effect size classifications

Statistic	Very small	Small	Medium	Large
Cohen’s *d*	< .20	.20	.50	> .80
Eta Squared (η2)		.02	.13	> .26
Hedges’ *g*		.20	.50	> .80
Rosenthal’s *r*		.10	.30	> .50

Cohen’s *d* and Eta Squared (Cohen, 1988); Hedges’ *g* (Hedges et al., 2012); Rosenthal’s *r* (Rosenthal, 1994)

Heterogeneity was explored visually using tables by comparing Hedges’* g* of studies and considering potential effect modifiers, including study protocol (e.g., duration and frequency of intervention, orientation, and compliance), study design and quality of studies. Studies rated as having a lower risk of bias were considered to hold more weight in the certainty of evidence, and strengths and limitations of the review were considered when drawing conclusions.

## Results

Table 4 presents the main characteristics of the studies.

**Table 4:** Study characteristics

First Author Year Country	Design	Population Setting Recruitment Inclusion Criteria	Sample characteristics *n* % Female Age (*M, SD*, Range)	Intervention characteristics *n* P/R-HRVBt Device Length & Frequency Orientation (O) Adherence Strategies (AS)	Control/comparator(s) *n* Length & Frequency	Intervention duration	Study completion rate %, *n*
van der Zwan (2015)^d^ Netherlands	RCT	Recruited stressed adults in Amsterdam and students from the University of Amsterdam Inclusion criteria: ≥ 17 on PSS	*n* = 76, range = 18–40 73% P/R-HRVBt group: *M* = 26.99, *SD* = 6.53, range = NR Physical Activity (PA) group: *M* = 25.28, *SD* = 4.42, range = NR Mindfulness meditation (MM) group: *M* = 26.32, *SD* = 5.03, range = NR	P/R-HRVBt group (*n* = 26) StressEraser handheld device Daily practices increasing in duration: week one: 10 min/day, week two: 15 min/day, and weeks three–five: 20 min/day O: One session, in person. Psychoeducation, practice, instructions AS: Self-monitoring logs, an implementation plan, daily text reminders. Payment incentive (50-euro voucher) was randomly allocated to 20 participants at the end of the study	PA group (*n* = 23), daily exercises increasing in duration: week one: 10 min/day, week two: 15 min/day, and weeks three–five: 20 min/day MM group (*n* = 27), daily exercises increasing in duration: week one: 10 min/day, week two: 15 min/day, and weeks three–five: 20 min/day	Five-week intervention	^a^88.2% (67/76)
Brinkmann (2020) Germany	RCT	Recruited employees of the Opera House and Theatre of Stuttgart, in Germany. Study information was sent to all employees Excluded participants who reported psychological illness or physical illness/heart rate or cortisol altering medications	*n* = 52 71% *M* = 43.27, *SD* = 10.45, range = NR	P/R-HRVBt group (*n* = 18) “Qiu” handheld device Daily 30 min practice O: One session, in person. Psychoeducation, instructions AS: Self-monitoring log. Two telephone monitoring calls and option of a refresher session for general or technical questions	Mindfulness-Based Intervention (MBI) (*n* = 15), daily 30-min practice Waiting List Control (WLC) (*n* = 19), no intervention	Six-week intervention	^a^96.7% (60/62)
Hsieh (2020) Taiwan	Cluster RCT (assigned eligible participants in the same hospital to any of the three arms)	Psychiatric ward nurses recruited from three hospitals Inclusion criteria: nurses who (1) had worked for at least three months, (2) had experienced any type of workplace violence exerted by patients or their families in the past 12 months Exclusion criteria: existing mental disorders before experiencing workplace violence (WPV)	*n* = 135 88.1% *M* = 35.61, *SD* = 8.81, range = 22–59	P/R-HRVBt (Referred to in the study as Smartphone-Delivered Biofeedback Training; SDBT) (*n* = 47) App—guided HRVBt self-training, containing guided shorter meditation practices and the processes of real-time HRVBt Once per week O: NR AS: Monitored online. Payment reward of approx. $12–14 for study completion	In-person Biofeedback training (BT) group (*n* = 49), 60-min session x once weekly Control group (*n* = 39), no intervention Control group was invited to receive the BT or SDBT intervention after the post-intervention measurement if they wished	Six-week intervention	^a^85% (135/159)
Son (2023) United States	CCT	Students recruited from a university in Texas Inclusion: 18 + and enrolled as students with a veteran	*n* = 24 8.3% *M* = 28.2, *SD* = 5.3, range = 19–40	P/R-HRVBt group (*n* = 10) App—mHealth Coaching: Biofeedback application Use daily “as needed” O: One session, in person. Measures collected, practice and instructions AS: Payment incentive at three points: $40 after 3rd online survey, $60 after 5th online survey, and US $100 after the exit interview	Control group (*n* = 14), instructed to do a breathing exercise “as needed”	20-week intervention	^a^91.6% (22/24)
Lemaire (2011) Canada	RCT	Physicians with work-related stress were recruited by email, post, and posters throughout the hospital Excluded if screened positive for severe depression on the PHQ-9	*n* = 40 42.5% Intervention group: *M* = 47.8, *SD* = 8.5, range = NR Control group: *M* = 44.8, *SD* = 8.2, range = NR	P/R-HRVBt group (*n* = 21) emWavePSR (Personal Stress Reliever) handheld device Practice on days zero to 28 for five-minutes at least three times daily. Trial extension (days 28 to 56) to use at own discretion O: One session, in person. Set up device, practice, instructions AS: Twice weekly phone contact to collect measures, record one three-minute Biofeedback session using emWavePC software and document adherence. Option of additional training between day 28 to 56	Control group (*n* = 19), no intervention Control group offered intervention at day 28 and instructed to use the device for five-minutes at least three times daily (without reinforcement or support)	Eight-week intervention	^b^NR
Deschodt-Arsac (2018) France	RCT	Athlete students recruited from the Sports Science department at the University of Bordeaux	*n* = 18 28% *M* = 20.5, *SD* = 1.5, range = NR	P/R-HRVBt group (*n* = 12) URGOfeel1 handheld device Practice for five-minutes twice a day (in the morning and evening) O: NR AS: Self-monitoring log	Control group (*n* = 6), no intervention	Five-week intervention	^b^NR
Kudo (2014) Japan	CCT	Mothers in early post-partum period with stress Inclusion: healthy mothers who had vaginal deliveries of a single infant, without medical complications Exclusion: mothers who habitually drank alcohol or smoked	*n* = 55 100% Intervention group: *M* = 30.5, *SD* = 5.7, range = NR Control group age: *M* = 33.4, *SD* = 6.6, range = NR Maternity blues was diagnosed in 16 mothers (29.1%)	P/R-HRVBt group (*n* = 25) StressEraser handheld device Daily use with a score of 30 points or more per session, and with enough sessions a day to achieve a total score of 100 points or more O: One session, in person. Set-up and practice AS: Self-monitoring log. Telephone monitoring call at two-weeks	Control group (*n* = 30), no intervention Participants who did not agree to use HRVBt served as the control group	Four-week intervention	^b^NR
Ratanasiripong (2015a) Thailand	RCT, stratified randomly for male and female	Students in the public nursing program at a public university in Thailand	*n* = 60 97% *M* = 34.05, *SD* = 7.61, range = 21–52	P/R-HRVBt group (*n* = 30) emWave Personal Stress Reliever Three times per day O: One session on set up and practice AS: Self-monitoring log	Control group (*n* = 30), no intervention	Four-week intervention	^b^NR
Ratanasiripong (2015b) Thailand	RCT	Students in the public nursing program at a public university in Thailand	*n* = 89 100% *M* = 19.27, *SD* = .56, range = 18–21	P/R-HRVBt group (*n* = 29) emWave Personal Stress Reliever device Three times per day O: Two sessions, in person. Set-up device AS: Self-monitoring log. A pay incentive (150 Baht) was given at the end of the study	Mindfulness mediation (MM) group (*n* = 29), practice MM three times per day Control group (*n* = 31), no intervention	Four-week intervention	^b^NR
Schuman(2019) United States	CCT, Pilot study	Adults recruited from Veterans Services, a southern US university and four counselling agencies Inclusion criteria: military-related PTSD with a score ≥ 15 on the PCL-5 Exclusion: nonmilitary PTSD, current or prior of psychosis, bipolar disorder, substance use, seizure disorder, diabetes, or cardiovascular disease, previous participation in an HRV study	*n* = 12 25% *M* = 36.16, *SD* = 10.45, range = 26–50 8/12 participants (66.6%) met DSM-5 criteria for PTSD	P/R-HRVBt group (*n* = 6) Phone App: MyCalmBeat Twice daily, 10–15 min each O: One session, in person. Measures collected, resonance frequency assessment to determine optimal breath rate, set up and instructions AS: Self-monitoring logs, weekly text message reminder, payment incentive for measures collected at each stage of study (pre, post, 16-week FUP)	Diaphragmatic breathing (DB) group (*n* = 6), DB twice daily, 10–15 min each practice After the four weeks, willing DB group participants received the P/R-HRVBt intervention (*n* = 5)	Four-week intervention	83.3% (10/12)
Ratanasiripong (2012) Thailand	RCT	Recruited 2nd year nursing students at a public nursing college in Thailand	*n* = 60 100% *M* = 19.27, *SD* = .61, range = 18–21	P/R-HRVBt group (*n* = 30) emWave Personal Stress Reliever handheld device Three times per day O: Two sessions, in person. Set up and how to use device AS: Self-monitoring log. A pay incentive (150 Baht) was given at the end of the study	Control group (*n* = 30), no intervention	Five-week intervention	^b^NR
Kizakevich (2019) United States	CCT, Pilot; app-evaluation study	Military personnel, veterans and civilian first responders in the US	*n* = 328 % NR *M* = NR, *SD* = NR, range = NR	Participants randomly assigned to one of four resilience training regiments Two of the four resilience training were paced breathing at five or six breaths per minute, each with P/R-HRVBt Biofeedback-Assisted Resilience Training (BART) application Five-minute practices three times a week for six-weeks, and twice a week thereafter for 12 months O: One session, in person. Set up app and practice AS: Daily text reminders. Online monitoring. Daily incremental incentives if all required measurements and training completed	Two of the four resilience training were paced breathing at five or six breaths per minute without the P/R-HRVBt Five-minute practices three times a week for six-weeks, and twice a week thereafter for 12 months	12 months	NR, study was still ongoing, but completion rates fell to 50% (164/328) by week two, 22.9% (75/328) at three months, 10% (33/328) at six months
Mensinger (2024) United States	Mixed-methods, single-arm pilot feasibility trial	Healthcare workers with elevated disordered eating scores during COVID-19 Various healthcare settings (e.g., metro/suburban/rural hospitals, long-term care, outpatient, EMT roles) across the U.S Email invitations to eligible CHAMPS registry LOCES > 2.6, English-speaking, Smartphone ownership, no serious medical contraindications for HRVB, no prior HRVB app experience	n = 28 89% *M* = 45.6, SD = 11.8, range = 28–65	n = 25 (fully enrolled) Photoplethysmographic (PPG) sensor and smartphone app (OptimalHRV) PPG sensor + OptimalHRV app 2-month intervention. Initial 4–5 min/session, building to 20 min/day HRVB Includes 5 baseline & 5 post-intervention 3-min HRV readings, and 1 resonance frequency assessment 60–90 min 1-on-1 Zoom training + resonance frequency assessment + 5 days baseline HRV readings Weekly Zoom/phone check-ins; email/text reminders; self-scheduling via Bookings app; motivational feedback; $75 gift card for completion	None (single-arm feasibility) N/A N/A	Approx. 2 months (with 2-week ramp-up and 6-week intervention)	82% (23/28)

*RCT* randomised control trial; *CCT* controlled clinical trial; *HRVBt* heart rate variability biofeedback training; *NR* not reported; *HRV* heart rate variability; *PSS* perceived stress scale (Cohen et al., 1983; Lee, 2012); *PCL-5* posttraumatic stress disorder checklist for DSM-5 (Blevins et al., 2015); *PHQ-9* patient health questionnaire (Kroenke et al., 2001); *LOCES* loss of control over eating scale-brief (Latner et al., 2014)

^a^Calculated based on information in full-text article

^b^Not calculated as information not available in full-text article and no response from authors upon request of supplementary information

^c^Confirmed by correspondence with author

^d^Information supplemented by de Bruin et al. (2016), who used the same study design and participants but published different outcome measures

### Study Characteristics

The included studies were published between 2011 and 2024 and were conducted in the following countries: USA (*n* = 4), Thailand (*n* = 3), Netherlands (*n* = 1), Japan (*n* = 1), France (*n* = 1), Taiwan (*n* = 1), Canada (*n* = 1), and Germany (*n* = 1).

The study designs are presented in Table 4 and studies were either RCTs (n = 8), control clinical trials (CCTs) (*n* = 4), or single-arm non-randomised (n = 1). Three of these studies were described as pilot studies. Three studies compared P/R-HRVBt to active comparators; one study had one active comparator and two had two active comparison groups. Six studies compared P/R-HRVBt to non-active control groups. Three studies had a combination of active and non-active control groups. Active comparators included diaphragmatic breathing, physical activity, mindfulness meditation, mindfulness-based intervention, practitioner-led HRVBt, and paced breathing. The interventions ranged from four weeks to 12 months, most lasting four weeks. Seven studies obtained measures at pre-and post-intervention, and five studies also measured outcomes at follow-up. Two studies collected adherence data weekly during the intervention phase and quarterly for up to 12 months. Quality ratings were derived using standardised appraisal tools; however, as blinding was not feasible in the included studies, this domain was omitted from the quality assessment ratings reported (Table 5).

**Table 5:** Study results

First Author Year Country	Quality assessment rating (excluding blinding)	Timing of assessments outcome measure(s)	Feasibility P/R-HRVBt Completion rate (%, *n*) Recruitment rate Adherence Dropout rate	Effectiveness (reported *p*, ES (*d,* η2, *r*), and calculated *g*)
van der Zwan (2015)^c^ Netherlands	Moderate	Pre-post intervention, six-week FUP Depression, Anxiety, and Stress (DASS) Sleep Quality (PSQI) Psychological Well-being (SPW) ^c^Attention (ACS) ^c^Executive Function (BRIEF-A) ^c^Mindfulness (FFMQ-SF) ^c^Self-compassion (SCS-SF) ^c^Worry (PSWQ)	88% (23/26) ^a^60% ^a^71% (P/R-HRVBt group: on average 25 days practice (*M* = 6.17 h, SD = 2.62). Practiced one third less than instructed ^a^11.8% (*n* = 9) (P/R-HRVBt: ^a^12%, *n* = 3)	P/R-HRVBt group: Significant reduction found pre to post intervention for DASS Stress (*p* < .05, *d* = − .33, ^a^*g* = − .32; S); DASS Anxiety (*p* < .05, *d* = − .40 ^a^*g* = − .30; S), SPW Psychological Well-being (*p* < .05, *d* = − .23; ^a^*g* = − .12; VS-S), ^d^PSWQ Worry (*p* < .05, *d* = − .36, ^a^*g* = − .21; S) Significant reduction found from pre to FUP for DASS Stress (*p* < .05, *d* = − .68, ^a^*g* = − .57; M); DASS Anxiety (*p* < .05, *d* = − .26, ^a^*g* = − .26; S), SPW Psychological Well-being (*p* < .05, *d* = − .06; ^a^*g* = − .05; VS), PSWQ Worry (*p* < .05, *d* = − .32, ^a^*g* = − .19; VS-S) Significant increase found pre to post intervention for ^c^ACS Attention (*p* < *.*05, *d* = .16, ^a^*g* = .11; VS), ^c^BRIEF-A Executive Function (*p* < .05, *d* = .19, ^a^*g* = .12; VS), ^c^FFMQ Mindfulness (*p* < .05, *d* = .68, ^a^*g* = .62; M), ^c^SCS Self-compassion (*p* < .05, *d* = .44, ^a^*g* = .25; S) Significant increase found pre to post for ^c^ACS Attention (*p* < .05, *d* = .01, ^a^*g* = .003; VS); ^c^BRIEF-A Executive Function (*p* < .05, *d* = .02, ^a^*g* = .003; VS), ^c^FFMQ Mindfulness (*p* < .05, *d* = .58, ^a^*g* = .60; M), ^c^SCS Self-compassion (*p* < .05, *d* = .16, ^a^*g* = .12; VS) NS reduction in DASS Depression or SPW Sleep Quality at post-intervention or FUP (*p* > .05) Between groups: NS differences between P/R-HRVBt, PA and MM groups for any of the ^c^outcomes (*p* > .05)
Brinkmann (2020) Germany	Moderate	Pre-post intervention, 12-week FUP Psychological: Chronic Stress (TICS) Stress Coping (SVF-120) Depression (BDI-II) Psychological Well-being (HEALTH-49) Mindfulness (FMI) Self-compassion (SCS) Physiological: HRV (SDNN, RMSSD: RSA and Short-term) Cortisol	100% (20/20) ^a^58.5% ^a^66% (P/R-HRVBt group practiced on average 19.74 (SD = 7.01) min/day ^a^3.3% (*n* = 2) (HRVB: ^a^0%, *n* = 0)	Psychological: Significant reduction in TICS Chronic stress pre-to-post intervention for P/R-HRVBt group (*p* = .01, ^a^*d* = − .44, ^a^*g* = − .43; S) NS differences found for P/R-HRVBt group at post-intervention or FUP on SVF Stress Coping, HEALTH Well-being, FMI Mindfulness, SCS Self-compassion, BDI Depression (*p* > .05) Physiological: Significant increase for HRV (SDNN short term measurement) pre to FUP (*p* = .01, ^a^*d* = .68, ^a^*g* = .66; M), and decrease for cortisol pre-to-post intervention (*p* = .016, ^a^*d* = − .81, ^a^*g* = − .80; L) for P/R-HRVBt group NS differences found for P/R-HRVBt group at post-intervention or FUP on any other measures of HRV (*p* > .05) Between groups: NS differences found between P/R-HRVBt, MBI and WLC groups (*p* > .05)
Hsieh (2020) Taiwan	Moderate	Pre-post intervention Psychological: Depression (CES-D) Resilience (RS) Occupational Stress (OSI-2) Physiological: HRV (SDNN, LF & HF power) Respiration Rate (RR)	^b^NR ^b^NR ^b^NR ^a^15% (*n* = 24) (SDBT 26.4%)	P/R-HRVBt (Referred to in this study as Smartphone-Delivered Biofeedback training; SDBT): Psychological: Significant reduction in CES-D Depression (*p* < .001, ^a^*d* = − 1.03, ^a^*g* = − 1.02; L), OSI Occupation Stress (*p* = .013, ^a^*d* = − .45, ^a^*g* = − .44; S), and increased RS Resilience (*p* < .001, ^a^*d* = .68, ^a^*g* = .67; M) from pre-to-post intervention Physiological: Significant reduction in respiration rates (*p* = .002, ^a^*d* = − .50, ^a^*g* = − .49; S-M), from pre-to-post intervention NS differences in SDNN (*p* = .75), LF (*p* = .52) and HF (*p* = .06) from pre-to-post intervention Between groups: NS differences between BT, SDBT and control group in CES-D depressive (*p* = .135) ^b^SDBT group (*p* < .001) had significantly higher resilience than did the control group (*p* = .36) but NS difference to the BT group (*p* = .355) ^b^SDBT group (*p* = .005) had a significantly lower occupational stress than the BT group (*p* = .32) and the control group (*p* = .06) There were no significant differences among the three groups in SDNN (*p* = .92), LF (*p* = .17), HF values (*p* = .69) and RR (*p* = .08)
Son (2023) United States	Moderate	Pre-post intervention + every two weeks for psychological measures only Psychological: Depression (PHQ-9) Anxiety (GAD-7) Physiological: Heart Rate (HR) Biofeedback App Engagement (relationship in App usage and PHQ-9 & GAD-7 scores	100% (10/10) ^d^90% 50% in HRVBt group 8% (*n* = 2) (HRVB: 0%, *n* = 0, Control: 8%, *n* = 2)	Psychological: NS differences found for the PHQ-9 Depression and GAD-7 Anxiety for the P/R-HRVBt group from pre-to-post (*p* > .05) Physiological: HR: ^b^Significant decreasing trend in HR for the six-minute windows after performing HRVBt for the P/R-HRVBt group (*p* < .001) Between groups: NS differences found between groups for the PHQ-9 Depression and GAD-7 Anxiety scores from pre-to-post intervention (*p* > .05). NR for HR outcome
Lemaire (2011) Canada	Weak	Day zero, 28 and 56 (pre-post, FUP) Psychological: Stress (merging two measures [15 items from the PSS + 25 items from the POQA-R]).Physiological: Heart Rate (HR) Blood Pressure (BP) Salivary Cortisol levels Adherence (good adherence defined as at least 15 min per day of self-reported use of device)	^b^NR ^b^NR ^a^28% (*n* = 6). Six out of 21 met the criteria for good adherence ^b^NR	Psychological: Significant reduction in stress from day 0–28 for the P/R-HRVBt group (*p* = .013, ^a^*d* = − .58, ^a^*g* = − .57; M) Significant reduction in stress from day 0–56 for the P/R-HRVBt group (*p* = .027, ^a^*d* = − .44, ^a^*g* = − .43; S) Physiological: NS changes in BP, HR, or salivary cortisol for P/R-HRVBt group over days 0–28, or over days 0–56 for the P/R-HRVBt group Between groups: There was a significant difference in stress score change between the P/R-HRVBt and the control group from day 0–28 (*p* = .048, ^a^*d* = .70, ^a^*g* = .66; M) NS changes in BP, HR, or salivary cortisol between groups over days 0–28 (*p* > .05) Due to the control group being exposed to the P/R-HRVBt intervention from day 28–56, there is no between group comparisons from day 28–56
Deschodt-Arsac (2018) France	Weak	Pre-post intervention (at exam one), five-week FUP (physiological only) and 12-week FUP (at exam two) Psychological: Anxiety (STAI) Physiological: HRV (RMSSD, HF power)	^b^NR ^b^NR ^b^NR ^b^NR	Psychological: NS difference in STAI Anxiety at post-intervention (*p* > .05).^b^Significant reduction in STAI Anxiety at 12-week FUP for P/R-HRVBt group (*p* < .001) Physiological: No within group effects were reported for the P/R-HRVBt group. However, it was noted there were no changes in HRV (RMSSD, HF power) from pre- to post-intervention and FUP. Whereas control group RMSSD and HF power reduced at post-intervention and FUP. Suggesting P/R-HRVBt helped maintain P/R-HRVBt group’s RMSSD and HF power in response to exam stressor Between groups: NR
Kudo (2014) Japan	Weak	Day four postpartum and one-month postpartum (pre-post intervention) Psychological: Postnatal Depression (EPDS) Physiological: HRV (SDNN, HF power, LF power) Heart Rate (HR)	^b^NR ^b^NR 80% (*n* = 20) and all mothers achieved at least one session per day ^b^NR	Psychological: Reduction in EPDS Depression for the P/R-HRVBt group from pre-to-post intervention (*p* < .001, ^a^*d* = − .76, ^a^*g* = − .74; M) Physiological: Reduction in HR (*p* = NR, ^a^*d* = − 1.70, ^a^*g* = − 1.64; L), and increase in SDNN (*p* < .01, ^a^*d* = 1.60, ^a^*g* = 1.52; L), HF power (*p* = NR, ^a^*d* = 1.11, ^a^*g* = 1.10; L), LF power (*p* = NR, ^a^*d* = 0.82, ^a^*g* = 0.81; L) for the P/R-HRVBt group from pre-to-post intervention Between groups: P/R-HRVBt group had significantly greater reduction in HR (*p* < .001, ^a^*d* = 1.40, ^a^*g* = 1.34; L), and increase in SDNN (*p* = .002, ^a^*d* = − .97, ^a^*g* = − .96; L), and EPDS (*p* < .001, ^a^*d* = 1.11, ^a^*g* = 1.10; L), compared to the control group at post-intervention NS differences in HF power (*p* > .05) and LF power (*p* > .05) between groups
Ratanasiripong (2015a) Thailand	Weak	Pre-post intervention Stress (PSS) State Anxiety (STAI) Depression (CES-D)	^b^NR ^b^NR ^b^NR ^b^NR	Significant reduction for PSS Stress (*p* < .05, *d* = − .01, ^a^*g* = − .04; VS), STAI-State Anxiety (*p* < .01, *d* = − .67, ^a^*g* = − .66; M), CESD Depression (*p* < .01, *d* = − .21, ^a^*g* = − .21; S) for the P/R-HRVBt group from pre-to-post intervention Significant differences found between the groups post-intervention (*p* = .02). P/R-HRVBt group had significantly lower STAI State Anxiety than the control group (*p* = .04, ^a^*d* = − .45, ^a^*g* = − .45; S). NS differences found between the groups for stress and depression
Ratanasiripong (2015b) Thailand	Weak	Pre-post intervention Stress (PSS) State Anxiety (STAI)	^b^NR ^b^NR ^b^NR ^b^NR	P/R-HRVBt group: NS reduction in PSS stress post-intervention (*p* > .05) Significant reduction in STAI-State Anxiety post-intervention (*p* = .006, η2 = .24, ^a^*g* = − .61; M) Between groups: NS differences were found between P/R-HRVBt, Mindfulness Meditation, and control group for PSS Stress post-intervention (*p* > .05) Significant differences found between the three groups for STAI-State Anxiety post-intervention (*p* = .006), with significantly lower scores for P/R-HRVBt compared with control group (*p* = .002, ^a^*d* = .79, ^a^*g* = .78; L)
Schuman (2019) United States	Weak	Baseline (BL) to four-week FUP (pre-post), 16-week FUP Psychological: PTSD (PCL-5) Physiological: HRV (SDNN, RMSSD) Acceptability (attrition, self-reported adherence)	100% (6/6) ^b^NR 70% > 17% (*n* = 2) (P/R-HRVBt + DB group: 0%, *n* = 0)	The study did not perform a statistical analysis of between-group differences. Instead, it combined the groups as a subsequent analysis once the control group had received the intervention Psychological: Reduction in PCL-5 PTSD from BL to four-week FUP (*p* < *.05*, *r* = − 2.50; L) and from BL to 16-week FUP (*p* < .01, *r* = .59, L) NS difference on the PCL-5 PTSD from four-week FUP to 16-week FUP for the P/R-HRVBt group (*p* > .05) Physiological: NS difference in SDNN or RMSSD from BL to 4-week FUP and NS difference in SDNN from BL to 16-week FUP (*p* > .05). Significant improvement in RMSSD from baseline to 16-week FUP (*p* < .05, *r* = .44, L)
Ratanasiripong (2012) United States	Weak	Pre-post intervention Stress (PSS) State-Anxiety (STAI)	^b^NR ^b^NR ^b^NR ^b^NR	NS reductions found for PSS Stress at post-intervention for the P/R-HRVBt group (*p* > .05) Significant reduction found for STAI State-Anxiety at post-intervention for the P/R-HRVBt group (*p* < .01, *d* = − .57, ^a^*g* = − .56; M) Between groups: P/R-HRVBt group had a significantly lower level of STAI State-Anxiety than control group post-intervention (*p* < .01, *d* = .68, ^a^*g* = − .67; M) NS group differences found for PSS Stress
Kizakevich (2019) United States	Weak	HRV (measured at rest, cognitive stress game, and during six-week training) Protocol adherence (measured weekly by incentive management plan over six-weeks and quarterly for 12 months)	NR—study not finished at time of reporting ^b^NR 63.1% (*n* = 207) for six-weeks. Following the six-week training period, compliance diminished below three trainings per week Adherence to self-report surveys = completion rates fell to 50.0% (164/328) by week two, then to 22.9% (75/328) at three months and 10.1% (33/328) at six months ^b^NR	NR—This evaluation study has not examined effectiveness
Mensinger (2024) United States	Weak	Pre-, mid-, and post-intervention assessments over a 2-month period HRV measured at rest (5-min ECG baseline) Adherence tracked daily via the app (with timestamped HRVB data), summarised at post-test Psychological: Disordered eating: LOCES, EDE-Q7 Stress and Resilience: PSS, SOC-R Mindful Self-Care and Body Attitudes: MSCS-B, BAS-2, IES-2 Interoceptive Awareness: MAIA-2 Physiological: HRV(RMSSD)	92% (23/25) 89% (25/28) 52% (13/25) met the goal of ≥ 67% adherence (≥ 28 days) 88% (22/25) met the goal of 33% adherence (≥ 14 days of ≥ 10 min practice) 14% (4/28)	Psychological: Significant reduction found for PSS at post-intervention (*p* < .001, *d* = 1.22, ^a^*g* = 1.18*;* L) Significant reduction found for Global Disordered Eating (EDE-Q7) at post-intervention (*p* < .001, *d* = 0.68, ^a^*g* = 0.66; M) Significant reduction found for Loss of Control Eating (LOCEs) at post-intervention (*p* < .001, *d* = 0.83, ^a^*g* = 0.81*;* L) Significant increase found for Intuitive Eating (IES-2) at post-intervention (*p* = .001, *d* = 0.63; ^a^*g* = 0.61*;* M) Significant increase found for Body Appreciation (BAS-2) at post-intervention (*p* = .001, *d* = 0.50, ^a^*g* = 0.49*;* M) Significant increase found for Sense of Coherence (SOC-R) at post-intervention (*p* = .030, *d* = 0.45, ^a^*g* = 0.44*;* M) Significant increase found for Mindful Self-Care (MSCS-B) at post-intervention (*p* < .001, *d* = 0.98; ^a^*g* = 0.95*;* L) Significant increases found for MAIA-2 subscales: Noticing (*p* = .001, *d* = 0.71; ^a^*g* = 0.69), Not Worrying (*p* = .017, *d* = 0.53), Attention Regulation (*p* < .001, *d* = 1.20, ^a^*g* = 1.16), Emotional Awareness (*p* = .005, *d* = 0.65, ^a^*g* = 0.63), Self-Regulation (*p* < .001, *d* = 1.02, ^a^*g* = 0.99), Body Listening (*p* < .001, *d* = 1.02, ^a^*g* = 0.99), Trusting (*p* = .002, *d* = 0.46, ^a^*g* = 0.45) NS trend for Not Distracting (*p* = .071, *d* = 0.45) Physiological**:** Significant increase in RMSSD during paced breathing post-intervention for the P/R-HRVBt group (*p* < .05) NS changes found in RMSSD at rest or during cognitive stress (*p* > .05)

*HRV* heart rate variability; *HRVBt* heart rate variability biofeedback training; *PA* physical activity, *MM* mindfulness meditation, *NR* not reported; *FUP* follow-up point; *NS* no significant; *SDNN* standard deviation of normal to normal; *RMSSD* root mean square of successive differences; *RSA* respiratory sinus arrhythmia (refers to the fluctuations of heart rate at the respiration frequency); *LF* low frequency; *HF* high frequency; *PTSD* post traumatic stress disorder; *DASS* depression, anxiety and stress scale (Nieuwenhuijsen et al., 2003); *PSQI* Pittsburgh sleep quality index (Buysse et al., 1989); *SPW* scales of psychological well-being (Ryff & Keyes, 1995); *ACS* attention control scale (Derryberry & Reed, 2001); *BRIEF-A* behaviour rating inventory of executive function (Roth et al., 2005); *FFMQ-SF* five facet mindfulness questionnaire—short form (Bohlmeijer et al., 2011); *SCS-SF* self-compassion scale (Neff, 2003); *PSWQ* Penn state worry questionnaire (Meyer et al., 1990); *TICS* Trier inventory for chronic stress (Petrowski et al., 2012); *SVF-20* Stressverarbeitungsfragebogen (Janke et al., 1997); *BDI-II* Beck-depression inventory (Hautzinger et al., 2009); *HEALTH-49* Hamburg modules for the assessment of psychosocial health in clinical practice (Rabung et al., 2009); *FMI* Freiburg mindfulness inventory (Walach et al., 2006); *CES-D* centre for epidemiological studies depression (Radloff, 1977); *RS* resilience scale (Friborg et al., 2006); *OSI-2* occupational stress indicator (Williams & Cooper, 1998); *PHQ-9* patient health questionnaire (Kroenke et al., 2001); *GAD-7* generalised anxiety disorder (Spitzer et al., 2006); *POQA-R* Personal and organizational quality assessment–revised questionnaire (Pipe et al., 2012); *STAI* state-trait anxiety inventory (Spielberger et al., 1971); *EPDS* Edinburgh postnatal depression scale (Cox et al., 1987); *PSS* perceived stress scale (Cohen et al., 1983; Lee, 2012); *PCL-5* posttraumatic stress checklist for DSM-5 (Blevins et al., 2015); *LOCES* loss of control over eating scale (Latner et al., 2014); *EDE-Q7* eating disorder examination questionnaire 7 (Grilo et al., 2015); *SOC-R* sense of coherence scale (Hittner, 2007; McGee et al., 2018); *MSCS-B *mindful self-care scale-brief (Hotchkiss & Cook-Cottone, 2019, 2023), *BAS-2* body appreciation scale-2 (Tylka & Wood-Barcalow, 2015); *IES-2* intuitive eating scale-2 (Tylka & Kroon Van Diest, 2013); *MAIA-2* multidimensional assessment of interoceptive awareness-2 (Mehling et al., 2018)

^a^Calculated based on information in full-text article

^b^Not calculated as information not available in full-text article and no response from authors upon request of supplementary information. VS, S, M, and L indicate very small, small, medium, and large effect sizes

^c^Information supplemented by de Bruin et al. (2016), who used the same study design and participants but published different outcome measures

^d^Confirmed by correspondence with author

Finally, all studies included either feasibility or effectiveness outcomes (Table 5). Five studies directly assessed feasibility and included one or more of the following: dropout, adherence, intervention completion rate or recruitment rates. Effectiveness was examined in 12 studies across differing psychological and physiological outcomes. Psychological outcomes were measured in all studies and included posttraumatic stress, depression, anxiety, stress, sleep, well-being, attention, executive function, mindfulness, self-compassion, worry, coping, body appreciation, self-care and resilience. Objective physiological stress markers, including HRV, heart rate, blood pressure, cortisol, and respiration, were measured in eight studies.

### Participant Characteristics

The total number of participants was 977, with sample sizes ranging from 12 to 328. Participants ranged from 18 to 65 years (reported by six studies). Eight studies comprised a majority female sample, with an average of 68.5% across studies (one study did not report on gender). Participants were recruited through volunteer and convenience sampling advertised in settings including Veteran centres, medical settings, universities, and the community. Most of the participants (*n* = 949) were from a non-clinical population and either did not meet clinical cut-off scores for anxiety or depression or were excluded if they had a mental health diagnosis or scored severely for depression. Four studies found high rates of mental health issues and stress among participants. One study specified a cut-off score of 15 or more on the PTSD checklist for the DSM-5 (PCL-5), and 75% of participants (*n* = 9) met the criteria for PTSD (Schuman & Killian, 2019); another study required participants to score 17 or more on the Perceived Stress Scale (PSS), indicating moderate to high-stress levels (van der Zwan et al., 2015), a third study found that 29.1% (*n* = 16) of mothers met the criteria for maternity blues (Kudo et al., 2014); and a fourth study enrolled healthcare workers with clinically significant disordered eating during COVID-19 (LOCES > 2.6) (Mensinger et al., 2024).

### HRV Biofeedback Characteristics

Across all included studies, the P/R-HRVBt intervention was completed using either a handheld device (*n* = 7) or a smartphone application (*n* = 6). The frequency and length of time spent practising P/R-HRVBt varied among the studies. For frequency, some studies instructed participants to complete two or three practices per day, while others instructed daily or weekly practices. Six studies instructed participants to practice the P/R-HRVBt for a set length of time, ranging from five-minute to 30-min practices. One study used a point system whereby points were accrued based on the time spent completing the intervention; participants had to score 30 points or more per session and reach 100 points per day. Two studies instructed participants to use the P/R-HRVBt daily “as required”. Four studies did not report the length of the P/R-HRVBt practices.

### Study Results

Table 5 presents the feasibility of each study and the effectiveness of P/R-HRVBt results. It displays effect sizes as reported by the author or calculated based on available data, using means, standard deviations, and sample sizes when the information was unavailable.

### Feasibility

Feasibility ranged across the included studies; out of the 13, recruitment rates were reported by three studies and ranged from 58.5 to 90%, with a mean of 69%. Five studies reported completion rates ranging from 83.3 to 92%, with a mean of 89%. The pilot app-evaluation study by Kizakevich et al. (2019) did not report a total completion rate because the study was ongoing but reported completion rates of 50% by week two, 22.9% at three months, and 10% at six months. The mean completion rate for the P/R-HRVBt groups was 96%. Adherence was reported by eight studies, with a mean of 60.01%. The range of adherence for the P/R-HRVBt groups was 28% to 80%. The dropout rates were based on five studies, with the average dropout rate being 11% and ranging from 3.3 to 17%. The average dropout rate for the P/R-HRVBt groups was 11%. Overall, the studies were variable in reporting feasibility but of those that did, there was generally good feasibility and adherence rates.

### Effectiveness

Twelve out of the 13 studies evaluated the effectiveness of the P/R-HRVBt intervention using either psychological measures (38%, *n* = 5) or a combination of psychological and physiological measures (62%, *n* = 7). The effectiveness across outcomes did not differ depending on the duration of the intervention or the length or frequency of which the participants were asked to practice P/R-HRVBt. The only exception was Son et al. (2023), who asked participants to practice P/R-HRVBt daily ‘as needed’ across a 20-week intervention and found minimal effects.

#### Psychological Outcomes

**Depression.** Six studies measured depression; four of these studies were of moderate quality (Brinkmann et al., 2020; Hsieh et al., 2020; Son et al., 2023; van der Zwan et al., 2015), and two were of weak quality (Kudo et al., 2014; Ratanasiripong et al., 2015a).

Three of the six studies demonstrated a significant reduction in depression symptoms for the P/R-HRVBt groups with varying effect sizes. However, no significant differences were found when comparing P/R-HRVBt to active comparator groups. In one study, P/R-HRVBt showed a greater reduction than the control group. While two studies reported depression scores at baseline (Kudo et al., 2014; van der Zwan et al., 2015), participants did not meet the clinical thresholds on the measures used. Consequently, these very tentative findings need to be considered in reference to subclinical levels of depression.

In Kudo et al. (2014), all participants did not meet the clinical cutoff score for postpartum depression of ≥ 10 on the Edinburgh Postnatal Depression Scale as the mean score on the scale was 4.60, and the standard deviation 1.99. Furthermore, in van der Zwan et al. (2015), participants just exceed the threshold criteria for anxiety but not for depression.

**Anxiety.** Seven studies examined the effectiveness of P/R-HRVBt in reducing general anxiety, worry, and PTSD symptoms from pre- to post-intervention. It is notable that only two of these studies report clinical cut-off data, with all veterans experiencing moderate PTSD symptoms in Schuman and Killian (2019) and a mean of 5.60 on the DASS scale with a standard deviation of 4.99 suggesting many participants just fell within the clinical range in van der Zwan et al. (2015). Two studies were of moderate quality (Son et al., 2023; van der Zwan et al., 2015), whereas the other five studies were of weak quality (Deschodt-Arsac et al., 2018; Ratanasiripong et al., 2012, 2015a, 2015b; Schuman & Killian, 2019), suggesting some caution should be exercised when considering these results.

Six of the seven studies (including the two with participants meeting the clinical threshold for anxiety) showed that P/R-HRVBt could improve anxiety symptoms, with small to medium effects. However, there is mixed evidence as to whether P/R-HRVBt is superior to active comparators or controls, and the evidence for sustained gains is limited, with only three studies including follow-up points.

**Stress.** Eight studies examined stress levels. The quality of the studies varied, with three of moderate quality (Brinkmann et al., 2020; Hsieh et al., 2020; van der Zwan et al., 2015) and five of weak quality (Lemaire et al., 2011; Mensinger et al., 2024; Ratanasiripong et al., 2012, 2015a, 2015b).

Six studies showed that P/R-HRVBt can reduce stress. However, there were mixed results when comparing P/R-HRVBt to active comparators and control groups, and only two studies included a follow-up point, so it is difficult to determine if improvements persist.

**Well-being.** Three studies, all scoring as moderate quality, investigated the effects of P/R-HRVBt on general well-being and outcomes associated with the positive aspects of maintaining good well-being, such as self-compassion, mindfulness, coping, sleep, and resilience.

Two studies found that P/R-HRVBt can improve resilience, general well-being, self-compassion, and mindfulness. However, when compared to active comparators, P/R-HRVBt was not superior.

**Other Psychological Effects.** One study examined the effectiveness of P/R-HRVBt on executive function and attention (van der Zwan et al., 2015). The study was of moderate quality and reported a significant improvement in executive function and attention from pre-to-post intervention, and from pre-intervention to follow-up, with very small effects (*d* = .01 to *d* = .19, respectively). However, no significant differences were found between groups at post-intervention or follow-up. Another study (Mensinger et al., 2024) examined the effectiveness of P/R-HRVBt on disordered eating, resilience, mindful self-care, body appreciation, intuitive eating, and interoceptive awareness. The study was of weak quality and found significant increases in mindful self-care (*d* = 0.98), body appreciation (*d* = 0.50), intuitive eating (*d* = 0.63), and interoceptive awareness subscales, along with a large reduction in perceived stress (*d* = 1.22).

#### Physiological Outcomes

**HRV.** Six studies measured HRV at pre- and post-intervention and reported on different indices, including the Standard Deviation of Normal to Normal (SDNN) intervals, the Root Mean Square of Successive Differences (RMSSD), Low-Frequency (LF) power, and High-Frequency (HF) power. Five studies were of weak quality (Deschodt-Arsac et al., 2018; Hsieh et al., 2020; Kudo et al., 2014; Mensinger et al., 2024; Schuman & Killian, 2019), while one was of moderate quality (Brinkmann et al., 2020).

Improvements in HRV demonstrated mixed evidence across studies. Two studies reported mixed effects, two found no effects, and one single-arm study (Mensinger et al., 2024) showed a non-significant 13% increase in HRV (RMSSD) with a small-to-medium effect size (*d* = 0.34).

**Other Physiological Stress Markers*.*** Five studies examined heart rate (HR), cortisol, respiration, and blood pressure. Three of these studies were of moderate quality (Brinkmann et al., 2020; Hsieh et al., 2020; Son et al., 2023), and two were of weak quality (Kudo et al., 2014; Lemaire et al., 2011).

Four studies showed significant differences in physiological stress markers for the P/R-HRVBt group, with one study showing a large between-group effect compared to a control group. However, two studies showed that P/R-HRVBt was not superior to active controls, and one did not examine between-group effects. Lastly, only one study included a follow-up point, making it difficult to determine any sustained gains.

## Discussion

This paper aimed to systematically review the feasibility and effectiveness of P/R-HRVBt for improving subclinical anxiety and depression and stress. Thirteen eligible studies were included, the majority of which recruited participants from non-clinical populations such as students, healthcare staff, and community volunteers who did not meet clinical thresholds for anxiety, depression, or stress. Only three studies specified clinical cut-off scores for mental health difficulties at screening, and only two had participants who met criteria.

All included studies examined either the feasibility or the effectiveness of P/R-HRVBt. One study examined feasibility only. The P/R-HRVBt intervention was completed remotely via a smartphone app or handheld device across all studies. Twelve studies measured the effectiveness of P/R-HRVBt with at least one psychological measure or a combination of psychological and physiological measures. These measures were taken pre- and post-intervention as a minimum.

### Summary of Evidence

#### Feasibility

Across the included studies, P/R-HRVBt appeared feasible, with dropout rates averaging 11%, comparable to HRVBt trials in chronic disease management (Fournié et al., 2021). However, these data were inconsistently reported (only five studies provided dropout rates) and may reflect recruitment bias, as participants were often self-selected and may have been highly motivated. Further, much heterogeneity exists in how adherence was monitored and reported between studies. Researchers’ adherence strategies varied, which included incentives, text reminders, or monitoring logs, but five studies did not report sufficient data to determine P/R-HRVBt compliance. Where data were available, average adherence was 60.01%. Interestingly, the rates were still similar when comparing the adherence rates of the studies that used payment incentives to those that did not. These findings align with the existing literature, which argues that there is mixed evidence regarding the association between the receipt of an incentive and adhering to a study protocol (Giles et al., 2014; Perski et al., 2022). Overall, feasibility seems promising in motivated, non-clinical populations, but research is required to determine whether engagement in P/R-HRVBt is sustained in clinical groups.

#### Effectiveness

All the included studies reported significant improvements in at least one outcome measure from pre- to post-intervention for P/R-HRVBt. Improvements were seen across psychological outcomes, including stress, subclinical anxiety and depression, clinical levels of anxiety, and physiological stress markers. However, substantial variation was found in effect sizes across the studies, ranging from very small to large, and it must be remembered that nine of the thirteen studies were rated as weak methodologically on the EPHPP. Of the studies that included follow-up measurements, these changes were generally shown to remain over time for psychological outcomes but were not consistently shown for the physiological stress markers. The lack of follow up data in most studies is problematic, so these findings are tentative at best.

A key finding across most of the studies was that P/R-HRVBt was not shown to be superior to active comparators at post-treatment and follow-up. This highlights that the active comparators of mindfulness-based interventions, mindfulness meditation, and physical activity also improved stress and mental health outcomes. This is a thought-provoking finding, as the results suggest that P/R-HRVBt is comparable to interventions that have been well-researched. While these comparator interventions are commonly used in the treatment of a range of mental health difficulties, the usefulness of P/R-HRVBt in clinical populations cannot be determined, as the vast majority of participants were from subclinical samples. Thus, observed changes should be understood as improvements in stress and subclinical difficulties rather than evidence of efficacy for diagnosed disorders. Nevertheless, given its low cost, accessibility, and acceptability among technologically literate users, P/R-HRVBt may have value for promoting wellbeing and stress reduction in the general population. Given the considerable demand on the UK health service, it is important that effective prevention strategies are identified and utilised where appropriate. However, future research needs to address the methodological weaknesses and lack of follow up data to determine if P/R-HRVBt training delivers enough to warrant further investment.

Further, several methodological issues regarding HRV could have contributed to the inconsistent HRV findings. Firstly, measuring HRV differed amongst the study protocols, with differences in recording duration, device used, and the HRV indices measured, making it difficult to interpret results across studies. Secondly, HRV outcomes may have been influenced by contextual factors such as caffeine intake, posture during measurement, or time of day, all of which can alter HRV readings (Koenig et al., 2013; Lalanza et al., 2023). As Grossman (2023) highlights, HRV is shaped by numerous external variables, raising questions about whether observed improvements represent durable changes in autonomic regulation or merely short-term, state-dependent effects. In this review, details of these contextual variables and of the P/R-HRVBt protocols were inconsistently reported, making it difficult to determine whether the findings reflect genuine intervention effects or methodological limitations.

Lastly, there was no clear trend across the studies suggesting that longer intervention durations, frequency, or length of P/R-HRVBt practice led to more effective outcomes. The only exception to this was the study by Son et al. (2023), who found no significant results across depression and anxiety outcomes. This could be because participants were asked to practice P/R-HRVBt daily ‘as needed’, with no information on how much time they needed to spend practising. It is, therefore, difficult to comment on the effectiveness of P/R-HRVBt in this study.

### Findings in Relation to Previous Evidence

Previous systematic reviews and meta-analyses have shown HRVBt to be effective for anxiety, stress, depression, physical health, and chronic disease management (Fournié et al., 2021; Goessl et al., 2017; Lehrer et al., 2020; Pizzoli et al., 2021). These reviews also found HRVBt to be as effective as active comparators. However, the need for more follow-up measurements across the studies was noted, meaning long-term efficacy for HRVBt remains uncertain.

These findings indicate that portable/remote HRVBt performs as well as in-person/laboratory HRVBt for the general population. Given the reduced cost and increased accessibility of portable/remote HRVBt this can be of benefit for both research and general population health applications.

### Evaluation of Included Studies

It is important to consider the quality of the studies when interpreting the findings. The quality assessment revealed methodological issues; all included studies received a weak assessment rating. When blinding was accounted for, four studies received a moderate rating. While this suggests higher quality research is needed to confirm current findings, it should be noted that many of the publications lacked information, which made it difficult to assess the quality of the studies.

Only five out of the 13 studies included a follow-up period (Brinkmann et al., 2020; Deschodt-Arsac et al., 2018; Lemaire et al., 2011; Schuman & Killian, 2019; van der Zwan et al., 2015). This is a significant limitation, as it is unclear whether treatment effects were maintained in more than half of the studies and potential delayed effects may have been missed, leading to an incomplete understanding of the intervention’s impact. Of the five studies with follow-up, only one (Lemaire et al., 2011) reported continued use of P/R-HRVBt during this period. It is unclear whether practice was maintained in the other four studies, whether participants wished to continue, or whether ongoing practice would have improved outcomes. This is important because practitioners aim to teach skills that can be sustained beyond the intervention to maintain results; therefore, further research should evaluate adherence during follow-up to provide greater clarity. Nonetheless, P/R-HRVBt could be a helpful short-term intervention for anticipated, time-bound situations, such as high-stress periods at work or exams. Caution is also needed when considering generalisability: although studies were conducted across eight countries, most used homogeneous, non-clinical, majority-female samples, limiting the applicability of findings to wider populations.

To summarise, quality ratings indicated that selection bias, blinding, withdrawal, and dropout were critical areas of weakness for many studies; therefore, future research should consider these, and the current findings should be interpreted cautiously when drawing conclusions.

### Evaluation of the Current Review

There were several strengths to this review. The search strategy was broad and thorough, with terms ensuring a comprehensive literature review and reflecting the wide range of psychological outcomes studied. Reference lists of included articles and past relevant systematic reviews were also searched. Research bias and methodological errors were minimised by using an independent reviewer for study selection and quality assessment. A sample bias correction enabled fairer comparison across smaller sample sizes, and searches were rerun at different timepoints to capture newly published papers.

While a limitation of this paper is the narrative synthesis, a meta-analysis was not possible due to the heterogeneity of the data, and to ensure rigour SWiM was utilised. Narrative synthesis is inherently subjective, creating a risk of bias from the reviewer’s interpretation, particularly as only one reviewer extracted the data (Campbell et al., 2019). To minimise this risk, a second independent reviewer was included, and the review followed the SWiM guidelines, which are designed for reviews of quantitative intervention effects when meta-analysis is not feasible (Campbell et al., 2020).

### Recommendations and Conclusions

With the increasingly important topic of the mind–body relationship and the use of technology in psychological interventions, this review provides an overview of studies using P/R-HRVBt. As the reviewed papers were of lower-quality studies, and there was heterogeneity across study designs, measures, and effect sizes, conclusions should be interpreted cautiously. Nevertheless, the findings suggest it is a feasible and effective intervention for improving stress, subclinical anxiety and depression in some of the populations described.

Based on the results of this review, P/R-HRVBt has been found to be as effective as the active comparators, such as mindfulness and physical activity, in a non-clinical sample. This has important implications for future practice. It suggests that P/R-HRVBt could be a useful alternative for individuals with busy schedules, as it can easily fit into their routine.

The review also found that P/R-HRVBt is feasible with low dropout rates for non-clinical populations. However, if this intervention is unsuitable for a particular population, the review has also shown that alternative therapies (the active comparators) are equally effective in reducing symptoms.

In summary, the review suggests that P/R-HRVBt is feasible and effective in improving stress, subclinical anxiety and depression. However, more research is needed using more rigorous methods, adequate follow-up periods and monitoring the use of P/R-HRVBt beyond the intervention phase to see if the results can be sustained over time.

## Data Availability

No datasets were generated or analysed during the current study.
